# The Effect of 17α-Ethynilestradiol and GPER1 Activation on Body and Muscle Growth, Muscle Composition and Growth-Related Gene Expression of Gilthead Seabream, *Sparus aurata* L.

**DOI:** 10.3390/ijms222313118

**Published:** 2021-12-04

**Authors:** Maria D. Ayala, Victoria Gómez, Isabel Cabas, María P. García Hernández, Elena Chaves-Pozo, Marta Arizcun, Daniel Garcia de la Serrana, Francisco Gil, Alfonsa García-Ayala

**Affiliations:** 1 Department of Anatomy and Comparative Pathological Anatomy, Faculty of Veterinary, Campus of Espinardo, University of Murcia, 30100 Murcia, Spain; cano@um.es; 2 Department of Cell Biology and Histology, Faculty of Biology, Campus of Espinardo, University of Murcia, 30100 Murcia, Spain; vga4@um.es (V.G.); icabas@um.es (I.C.); piligar@um.es (M.P.G.H.); agayala@um.es (A.G.-A.); 3 Centro Oceanográfico de Murcia, Instituto Español de Oceanografía (IEO-CSIC), Puerto de Mazarrón, 30860 Murcia, Spain; elena.chaves@ieo.es (E.C.-P.); marta.arizcun@ieo.es (M.A.); 4 Department of Cell Biology, Physiology and Immunology, Faculty of Biology, University of Barcelona, 08028 Barcelona, Spain; garciadelaserrana@ub.edu

**Keywords:** EE_2_, GPER1, body growth, muscle cellularity, muscle growth regulator genes

## Abstract

Endocrine-disrupting chemicals include natural and synthetic estrogens, such as 17α-ethynilestradiol (EE_2_), which can affect reproduction, growth and immunity. Estrogen signalling is mediated by nuclear or membrane estrogen receptors, such as the new G-protein-coupled estrogen receptor 1 (GPER1). The present work studies the effect of EE_2_ and G1 (an agonist of GPER1) on body and muscle parameters and growth-related genes of 54 two-year-old seabreams. The fish were fed a diet containing EE_2_ (EE_2_ group) and G1 (G1 group) for 45 days and then a diet without EE_2_ or G1 for 122 days. An untreated control group was also studied. At 45 days, the shortest body length was observed in the G1 group, while 79 and 122 days after the cessation of treatments, the shortest body growth was observed in the EE_2_ group. Hypertrophy of white fibers was higher in the EE_2_ and G1 groups than it was in the control group, whereas the opposite was the case with respect to hyperplasia. Textural hardness showed a negative correlation with the size of white fibers. At the end of the experiment, all fish analyzed in the EE_2_ group showed a predominance of the gonadal ovarian area. In addition, the highest expression of the *mafbx* gene (upregulated in catabolic signals) and *mstn2* (myogenesis negative regulator) was found in EE_2_-exposed fish.

## 1. Introduction

Endocrine-disrupting chemicals (EDCs) encompass both naturally produced compounds, such as estrogens and androgens, and a wide range of industrial chemicals released from many sources into the environment. These compounds are capable of altering not only the function of the endocrine system but also modulate reproduction, immunity and growth, among other things [1,2,3,4]. A synthetic estrogen, 17α-ethynilestradiol (EE_2_) is used in oral contraceptives and hormone replacement therapy. Exposure to EE_2_ causes alterations in reproductive capacity [5,6,7,8,9,10,11,12,13] and sex differentiation in fish [14,15,16]. The disruptive effects of this compound vary according to dose, time of exposure, age and the stage in the reproductive cycle of the treated individuals [5,6,7,8,9,10,11,12,13,14,15,16].

Estrogen signalling is carried out by nuclear and membrane estrogen receptors, which mediate genomic actions and non-genomic rapid effects, respectively. The G protein-coupled estrogen receptor (GPER1) is a membrane estrogen receptor that binds E_2_ (17β-estradiol) and other estrogens. The first GPER1-selective ligand identified was G1 [17,18], which has been extensively used to explore the biological significance of GPER1 in different tissues and organs [19].

The gilthead seabream (*Sparus aurata* L.) is a protandrous hermaphroditic fish species that develops as a functional male for the first two years, after which 40% of a given population go on to develop as females during the third year [20]. Therefore, the gonads of this species present two areas, a testicular and an ovarian area, which are macroscopically distinguishable [21]. The testicular area becomes functional during the first two reproductive cycles, while the ovarian area remains formed with immature oocytes or early vitellogenic oocytes. However, the ovarian area proliferates at the end of the first and second reproductive cycles [21].

Previously, the influence of EE_2_ on the reproductive parameters of functional males of this species has been studied by our team [22]. EE_2_ exposure in male specimens in the second reproductive cycle was able to significantly increase hepatic transcript levels of the gene coding for vitellogenin (*vtg*) and plasma levels of Vtg, causing a robust endocrine disruption followed by a return to basal levels once exposure ceased. The disruption of the reproductive endpoints studied lasted for a long time, even when exposure to EE_2_ had cessed, resulting in a failure of the natural sex change previously mentioned [20]. Moreover, the influence of different concentrations of EE_2_, as well as the contribution of GPER1, on these effects has also been demonstrated previously by our team [23]. However, given that the effect of these compounds depends on different factors [5,6,7,8,9,10,11,12,13,14,15,16], exposure and recovery periods different from those applied in the aforementioned studies have been examined in the present work to expand knowledge of the effect of these compounds on the second reproductive cycle of seabream specimens.

So far, studies of the influence of natural or synthetic steroids in fish muscle growth dynamics are scarce and they have been carried out mainly in salmonids [24,25,26,27,28,29,30]. No studies are available of the effects of EE_2_ and GPER1 contributions with respect to fish muscle growth. The studies in salmonids show a catabolic effect of natural estrogens on fish muscle, with reductions in growth and also of fillet yield. It has been previously reported that E_2_ caused a reduction in the growth of female trout, *Oncorhynchus mykiss,* during sexual maturity. These effects were associated with a redirection of nutrients to gonadal development at the expense of muscle growth [24]. However, these steroid responses vary among fish species, as E_2_ can promote growth in some fish, e.g., yellow perch, *Perca flavescens* [25]. Some studies in fish indicate anabolic effects of androgens on muscle growth [26,27], whereas other studies have shown that testosterone induces a catabolic response in muscle [28,29]. Therefore, in fish, the effects of androgens on protein growth in skeletal muscle may be dose- or developmental stage-dependent. In contrast, in mammals, it has been observed that steroids (estrogens and androgens) exert an anabolic effect, increasing protein accretion and muscle mass [30].

The mechanisms of fish muscle growth (hypertrophy and hyperplasia) require the activation of specific muscle cells (myoblasts) which can either form new fibers (hyperplasia) or fuse with existing fibers (hypertrophy) [31]. Both muscle growth and development are modulated by an intricate network of transcription factors, signaling pathways, non-coding RNAs and endocrine factors, integrating biotic and abiotic inputs and modulating muscle plasticity [32]. Hyperplasia (generation of new fibers) determines the total number of fibers in fish muscle and so influences their future growth capacity and architecture [31,32]. Hypertrophy refers to the increase in size of pre-existing fibers and, while both growth mechanisms (hypertrophy and hyperplasia) can happen simultaneously during the early life stages of fish, hyperplasia stops at advanced stages of fish growth while hypertrophy can continue to affect growth [31,32].

It is known that myogenic regulatory factors (MRFs), a family of transcription factors, regulate the progression of the myogenic program from lineage determination (*Myod, Myf5*) to fiber differentiation (*MyoG, Myf6*), acting together with other genes able to regulate proliferation (e.g., *Pcna*) or fusion (*Dock5*, *Cav3*, *Plekho1* and *Tmem8c*).

Muscle growth is highly dependent on the balance between protein synthesis and degradation. Various factors regulate these two processes, such as growth factors (e.g., *Igf1*, *Igf2* or *Gh1*) and nutrients (mainly amino acids). Protein synthesis is strongly regulated by the *Pi3k*/*Akt*/*mTor* pathway, which is activated by the influence of growth factors as well as nutritional factors [33,34]. Protein degradation in muscle follows three main pathways: proteasome, lysosomal and calpains, which increase muscle atrophy when they are activated [31,35]. On the other hand, muscle parameters (hypertrophy and hyperplasia) affect meat quality, mainly the texture of the fish fillet [36,37,38]. Hence, the aim of the present work was to determine the influence of EE_2_ and GPER1 on body and muscle growth of protandrous gilthead seabream during their second reproductive cycle. To that end, gilthead seabreams were exposed to EE_2_ or G1 and analyses were carried out at day 45 of treatment and 79 and 122 days after treatment cessation to evaluate short and long-lasting effects, respectively. Since muscle cellularity influences meat quality, muscle composition and fillet texture were analyzed throughout the experiment. Moreover, genes related to muscle development (*myod1* and *mstn2*) and protein catabolism (*mafbx* and *capn1*) were determined.

## 2. Results

### 2.1. Body Parameters, Cumulative Intake and Survival

#### 2.1.1. Body Parameters

After 45 days of treatment (first stage), the mean values of the total body weight (BW) and the gutted weight (GW) were higher in the C group than in the treated groups (EE_2_ and G1 groups) (Table 1). The body length (BL) showed the lowest values in the G1 group (*p* < 0.05). In relation to the condition factor (CF), this was significantly lower in the EE_2_ group than in the G1 group, whereas the C group did not show significant differences compared to other groups. The hepatosomatic and gonadosomatic indices (HSI and GSI, respectively) did not show significant differences among groups.

Seventy-nine days after treatment cessation (second stage), body growth values (BW, GW and BL) gradually increased in the three groups in comparison with the first stage, even though the increase was only statistically significant in the C and G1 groups (Table 1). When comparing body values among the three groups at this stage, we can observe that both BW and GW were significantly lower in the EE_2_ group than in the other groups (*p* < 0.05) (Table 1). Similarly, BL was also lower in the EE_2_ group, and this change was statistically significant in comparison with the C group. The CF was also lower in the EE_2_ group but not statistically significant. The HSI decreased significantly at this stage in comparison with the first stage in the three groups, while the values of the GSI did not show significant differences (Table 1). When comparing these values among the three groups at this stage both indices (HSI and GSI) were similar in the three groups (*p* > 0.05).

At the end of the experiment (122 days after treatment cessation, third stage), the body growth values (BW, GW and BL) increased significantly in all the groups (Table 1). When comparing the mean values among the three groups at this stage, we found that the BW, GW, BL and CF values were lower in the EE_2_ group than in the other groups (Table 1). The HSI values decreased significantly at this stage in comparison with the HSI values at the beginning of the experiment in the three groups, whereas the values of the GSI did not show significant variations. However, when comparing the mean values of both indices (HSI and GSI) among the three groups at the end of the experiment, the lowest values of HSI were observed in EE_2_, and the highest values of GSI were observed in C and EE_2_ (*p* > 0.05) (Table 1).

#### 2.1.2. Cumulative Intake and Survival

**Figure 1:**
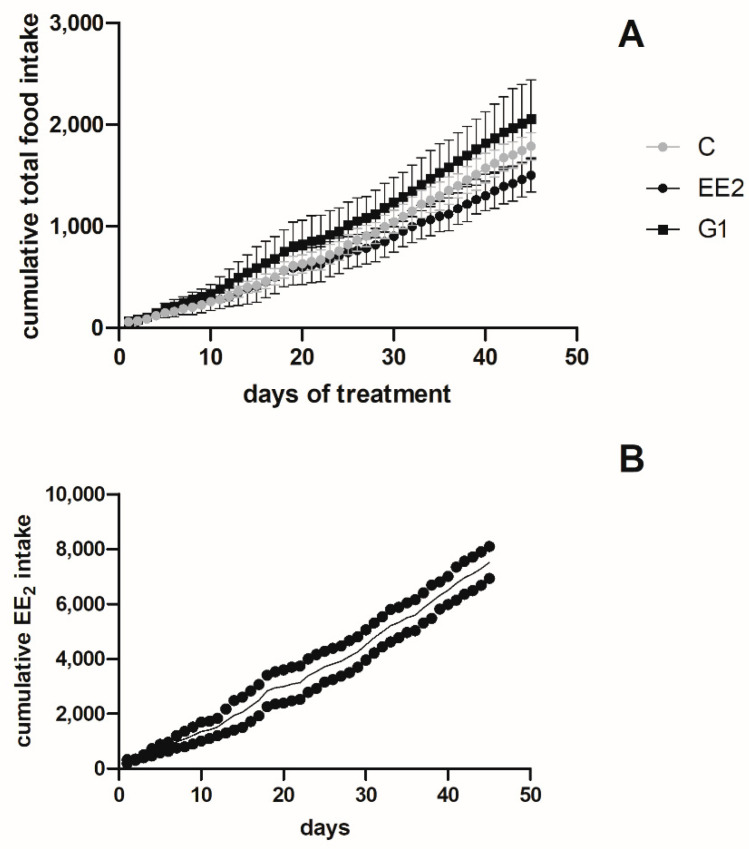

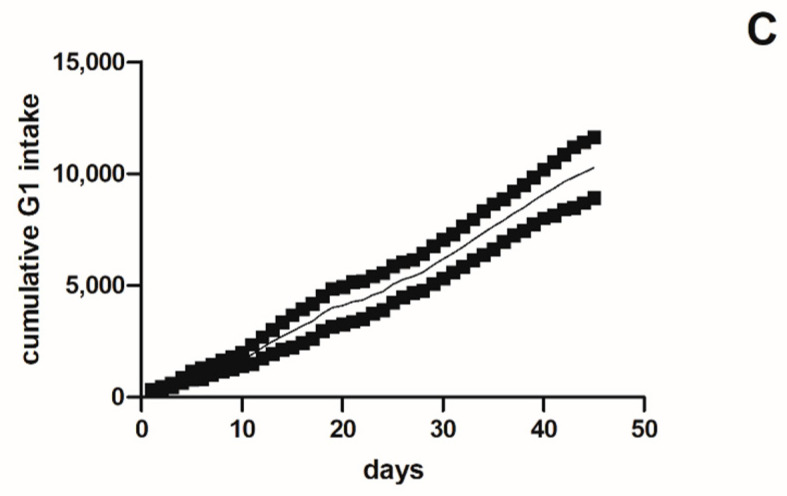
(**A**) Total cumulative food intake (g) of the different experimental groups for the 45 days that the treatments lasted. The data are represented as the mean value ± SEM of the duplicate tanks for each treatment. (**B**) The amount of EE_2_ ingested (µg) by the fish in each experimental tank is represented by circles, while the mean values of both tanks are represented by a black line. (**C**) The amount of G1 ingested (µg) by the fish in each experimental tank is represented by squares, while the mean values of both tanks are represented by a black line.

Dietary intake was measured in the three groups (C, EE_2_ and G1) and the cumulative total food intake was calculated for the 45 days that the treatments lasted. No statistically significant differences were observed among groups (Figure 1A). Thus, when the cumulative amount by each tank was analyzed, no differences were observed between the replicates (Figure 1B), neither for the EE_2_ nor G1 cumulative intake (Figure 1C).

Survival was 100% in the C and G1 groups, whereas it was 83.0 ± 6.7% (mean ± SD) in the EE_2_ group at the end of the experiment.

#### 2.1.3. Fish Gonad Structure

After 45 days of treatment and 79 days of recovery (first and second stages, respectively), the gonads of all the specimens showed predominantly testicular areas (100% T fish). However, 122 days after the end of exposure (third stage), a change in the proportion of testicular and ovarian areas of the fish gonads was observed, 66.67% of the studied specimens in the C group (4/6 specimens) showing an ovarian area predominance (Ov fish), whereas G1 and EE_2_ showed 50% (3/6 specimens) and 100% (6/6 specimens) of Ov fish, respectively.

**Figure 2:**
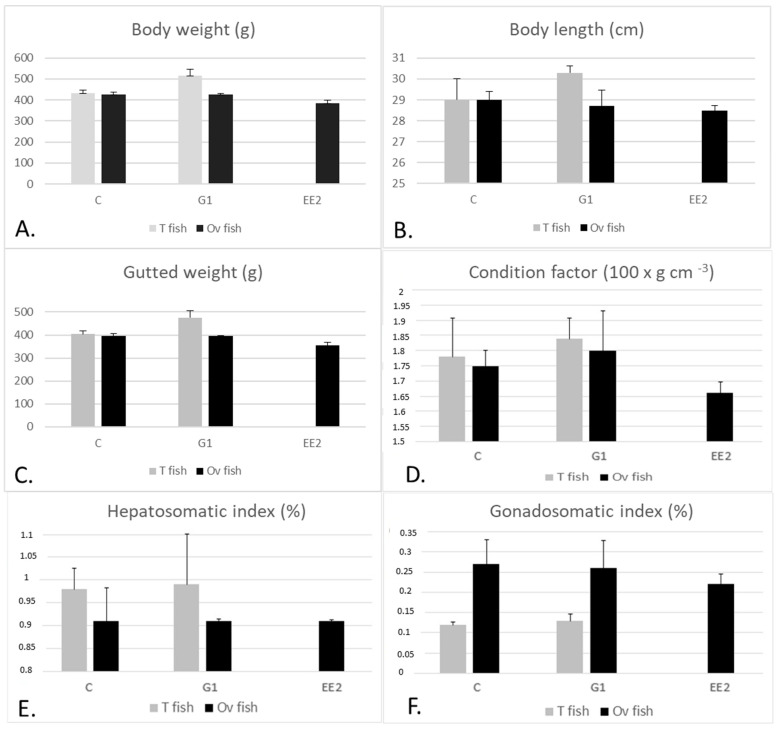
(**A**–**F**) Mean values (+SEM) of body weight, body length, gutted weight, condition factor, hepatosomatic and gonadosomatic indices, respectively, in T and Ov specimens of C, G1 and EE_2_ groups at the end of the experiment. The fish in the EE_2_ group were 100% Ov fish at the end of the experiment and therefore there are no data for T fish in this group. No statistically significant differences were observed among the different groups.

When comparing the body values of T fish between the C and G1 groups, the differences observed were not statistically significant, although T fish in the C group were smaller than T fish in the G1 group (Figure 2). Ov fish in C were similar to Ov fish in G1. On the other hand, when comparing the body parameters of T specimens versus Ov specimens from the three groups, we observed that most of the parameters were slightly higher in T than in Ov specimens (*p* > 0.05), except for GSI, which was higher in Ov than in T specimens. When comparing the values among Ov specimens of the three groups, the smallest body values were usually observed in the EE_2_ group (*p* > 0.05) (Figure 2).

### 2.2. Muscle Parameters

**Figure 3:**
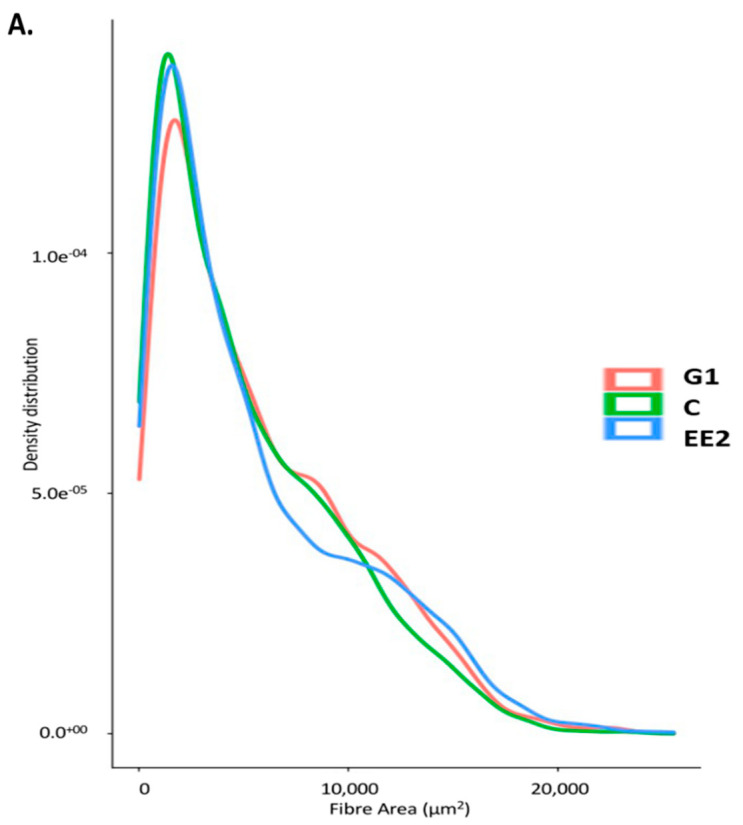

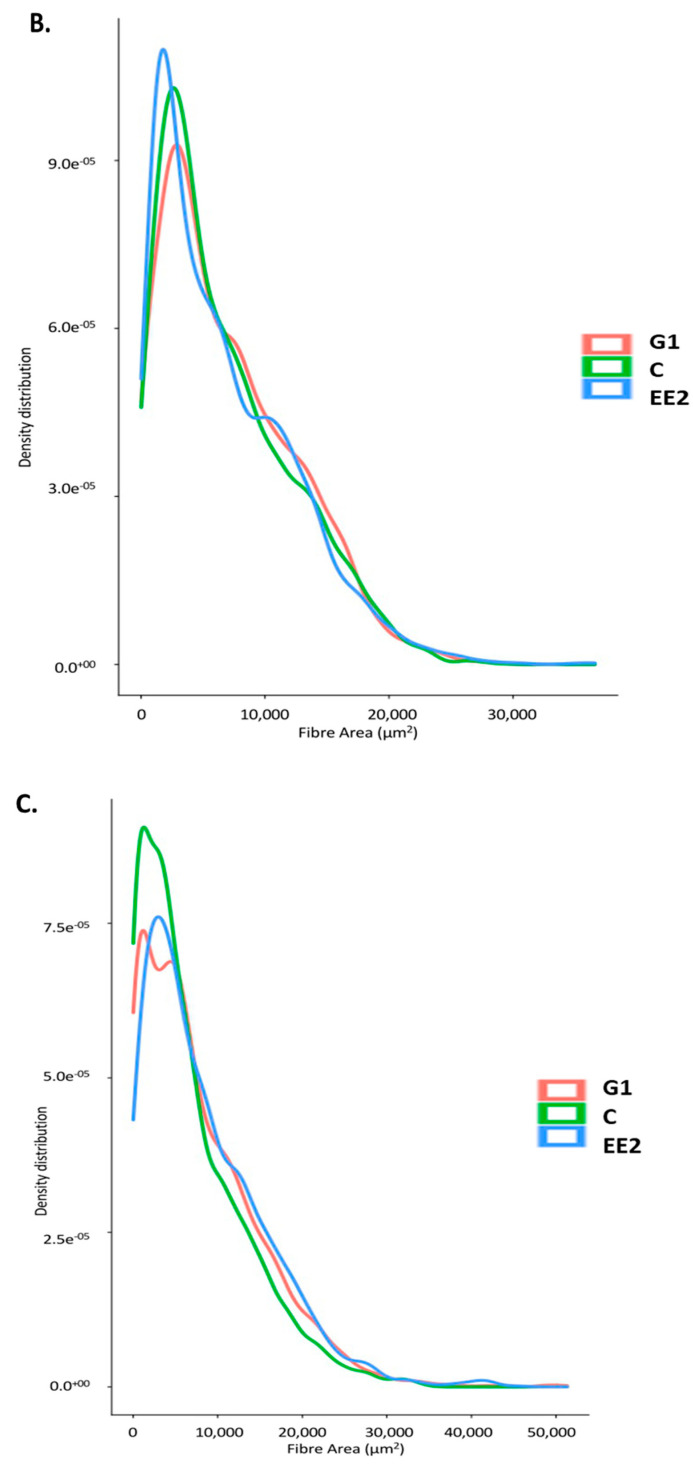
Size distribution of white muscle fibers (µm^2^) of the three experimental groups at the first stage (on day 45 of treatment) (**A**), at the second stage (79 days after treatment cessation) (**B**) and at the third stage (122 days after treatment cessation) (**C**). Red, green and blue lines correspond to the G1, C and EE_2_ groups, respectively.

After 45 days of treatment, the cross-sectional area of white (W) and red (R) muscle and total myotome values did not show significant differences among the three groups (Table 2). In relation to the muscle cellularity of the white muscle, the largest size of white fibers was found in the G1 group, followed by the EE_2_ group. The C group showed the smallest size, even though these differences were not statistically significant (*p* > 0.05) (Table 2). The number and density of white muscle fibers showed the opposite tendency to that observed for fiber size, such that these were higher in the C group, followed by the EE_2_ group, whereas the G1 group showed the lowest value of hyperplasia, and this was correlated with a lower percentage of small fibers (fibers of new generation) in this group compared with C and EE_2_ group (Table 2; Figure 3A).

After 79 days of treatment cessation, the values of W, R and the total myotome significantly increased (*p* < 0.05) in all the groups (Table 2). When the three groups were compared with each other at this stage, we observed that the mean values of the cross-sectional area of the W, R and total myotome did not show significant differences among them (*p* > 0.05) (Table 2). However, the values were higher in the C and G1 groups than in the EE_2_ group. In relation to muscle cellularity, muscle growth mainly increased by hypertrophy in the three groups (Table 2). This increase in hypertrophy was significant in the C and G1 groups. On the contrary, the number of fibers did not significantly increase in any group in this stage. When comparing the three groups with each other at this stage, we found that the lowest value of the area of the white fibers was observed in the C group, followed by the EE_2_ group, whereas the G1 group showed the highest values (*p* > 0.05) (Table 2). In contrast, the number of white fibers and the fibrillar density were higher in the C group, followed by the EE_2_ group, the G1 group showing the lowest values (*p* > 0.05) of hyperplasia and the lowest percentage of fibers of new generation (Table 2, Figure 3B).

At the end of the experiment (122 days after treatment cessation), muscle growth significantly increased in all the groups. When comparing the three groups with each other at this stage, the highest values of W, R and total myotome were obtained in the C group, followed by the G1 group. The EE_2_ group showed the lowest values (Table 2). In relation to muscle cellularity, the C and G1 groups showed increased fiber generation, and the increase was significant in the C group (Table 2). In contrast, hyperplasia did not increase in the EE_2_ group in this stage. On the other hand, hypertrophy increased significantly in the EE_2_ group, while this parameter remained constant in the other groups. When comparing muscle cellularity among the three groups at this stage, the largest size of white muscle fibers (area and minimum diameter) was found in the EE_2_ group, followed by the G1 group. The C group showed the lowest values of this parameter (Table 2). In contrast, the highest values of the number and density of white muscle fibers were reached in the C group, followed by the G1 group. The EE_2_ group showed the lowest values of both parameters. The highest hyperplasia values in the C group were parallel to the generation of a higher percentage of new fibers in this group (Figure 3C).

#### 2.2.1. Muscle Parameters According to the Gonad Structure

**Figure 4:**
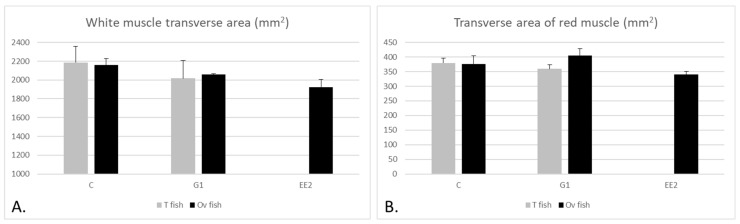
(**A**,**B**) Mean (+SEM) of the transverse area of the white (W) and red (R) muscles, respectively, in T and Ov fish in the three groups at the end of the experiment. The fish studied in the EE_2_ group were 100% Ov fish at the end of the experiment and therefore there are no data for T fish in this group. No statistically significant differences were observed among the different groups.

When comparing the W values of the T and Ov fish in the C group with the T and Ov fish in the G1 group, no significant differences were observed, even though the values were higher in the C group compared to the G1 group (Figure 4A). On the other hand, R was similar between T fish in the G1 group and T fish in the C group, but it was larger in Ov fish in the G1 group than in Ov fish in the C group (*p* > 0.05) (Figure 4B). On the other hand, when comparing W and R among the Ov specimens of the three groups, the lowest values were observed in the EE_2_ group (*p* > 0.05) (Figure 4).

**Figure 5:**
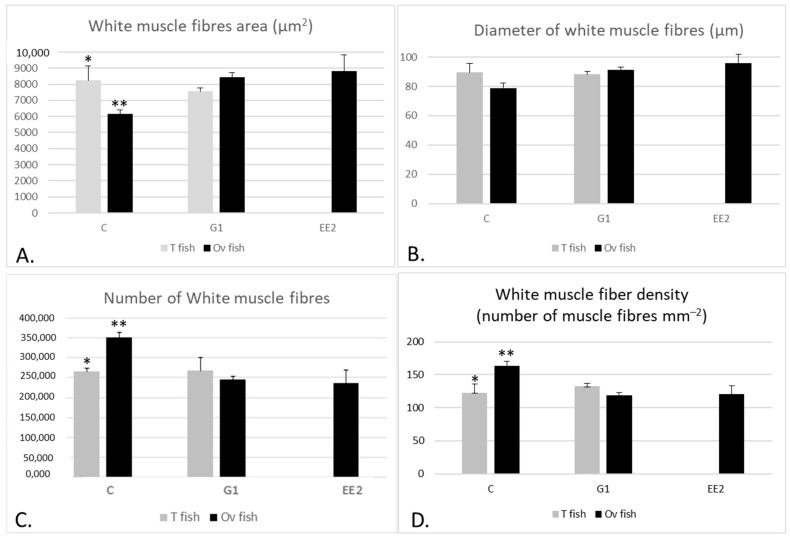
(**A**–**D**) Mean (±SEM) values of the size of white fibers (area and minimum diameter) and the number and density of the white muscle fibers, respectively, in male and female specimens from the three groups at the end of the experiment. The fish studied in the EE_2_ group were 100% Ov fish at the end of the experiment and therefore there are no data for T fish in this group. * Denotes significant differences (*p* < 0.05) between T and Ov fish in the C group for the area, number and density of the white muscle fibers. ** Denote significant differences (*p* < 0.05) between Ov fish in the C group and Ov fish in the EE_2_ and G1 groups for the area, number and density of the white muscle fibers.

In relation to muscle cellularity (hypertrophy and hyperplasia), the area of the white muscle fibers was significantly higher in T than in Ov fish in the C group (*p* < 0.05), whereas the number and muscle fiber density showed the contrary tendency (*p* < 0.05) (Figure 5). In contrast, the Ov fish in the G1 group showed larger sizes of white muscle fibers than T fish, whereas the number and muscle fiber density of white fibers was lower in Ov than in T specimens in this group. However, in the G1 group, these differences were not significant.

When comparing muscle cellularity of the Ov fish among the three groups, the largest size of white muscle fibers was observed in the EE_2_ group, followed by the G1 group, the C group showing the lowest values (*p* < 0.05) (Figure 5A,B). The number of fibers showed the opposite tendency to that described for the size of fibers (*p* < 0.05) (Figure 5C,D).

#### 2.2.2. Histochemical Characterization of the Muscle Fibers

**Figure 6:**
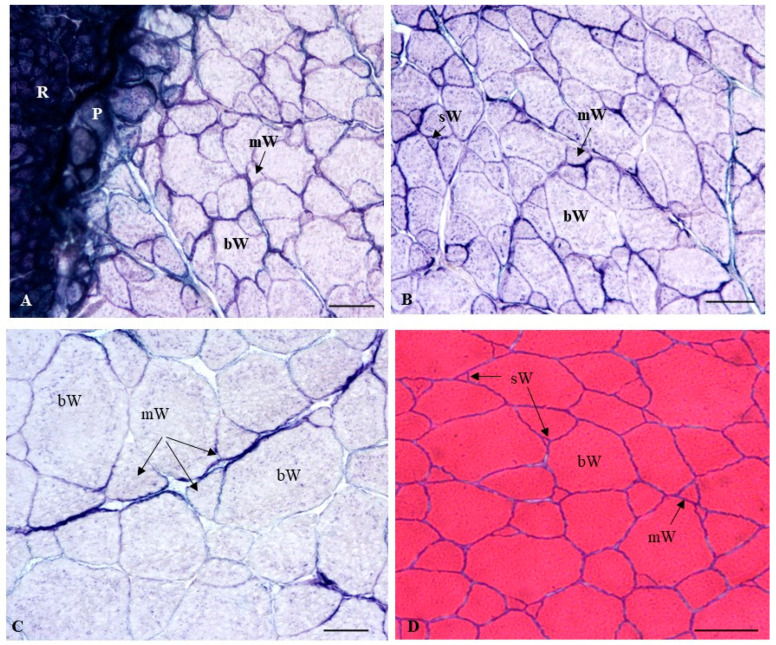

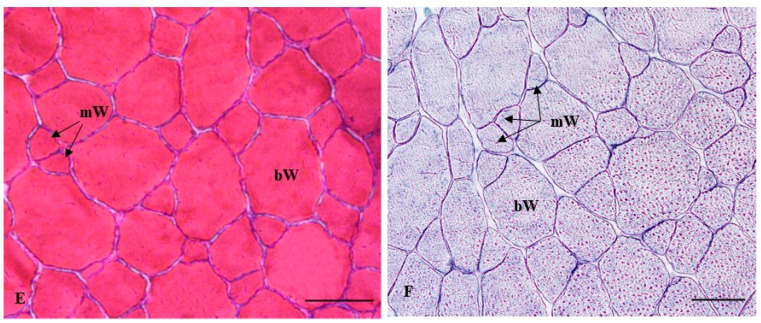
Transverse sections of the myotome of seabream from the C group (**A**,**B**) and the EE_2_ group (**C**) at the first stage (on day 45 of treatment). (**D**) corresponds to T fish in the C group at the end of the experiment (122 days after treatment cessation). (**E**,**F**) correspond to Ov fish in the EE_2_ group and G1 group, respectively, at the end of the experiment. NADH-TR (**A**–**C**,**F**) and hematoxylin-eoxin (**D**,**E**) stains. W, R, P: white, red and pink muscles, respectively; bW, mW and sW: big, medium and small white muscle fibers, respectively. Bars: (**A**,**B**): 100 µm; **C**: 50 µm; (**D**–**F**): 500 µm.

In relation to the histochemical profile of the muscle fibers, it was observed that red muscle showed an intense blue color after staining with the NADH-TR technique, indicating its typical oxidative (aerobic) metabolism (Figure 6A). In contrast, the large white fibers (mature white fibers) appeared unstained (white color), as corresponds with their typical glycolytic (anaerobic) metabolism. However, the white fibers of medium- and small-sized (immature white fibers) showed a weak bluish coloration, thus indicating that they still sustained a certain amount of oxidative activity prior to their complete maturation in which their metabolism would have become glycolytic (Figure 6A–C). Thus, in the first and second stages, since the white muscle fibers of the treated groups (EE_2_ and G1) presented greater hypertrophy than the C group, the glycolytic potential of the white muscles in the EE_2_ and G1 groups was higher than that observed in the C group (Figure 6A–C). At the end of the experiment, the histochemical profile of the muscle fibers was similar to that observed in the previous stages, with higher glycolytic activity in the larger white fibers (Figure 6D–F). Thus, in the last stage, since the greatest hypertrophy values were reached in the EE_2_ and G1 groups, as well as in the T fish in the C group, the glycolytic potential of these groups was higher than that observed in the Ov fish in the C group.

### 2.3. Muscle Composition and Textural Hardness

After 45 days of treatment, the percentage of muscle protein was similar in the three groups. The lowest values of muscle fat were observed in the EE_2_ group (Table 3). Regarding the textural firmness values of white muscle, these were lower in the G1 group than in the other groups, although not statistically significant (*p* > 0.05) (Table 3).

After 79 days of treatment cessation, the muscle protein values were similar to those observed in the first stage, except in the EE_2_ group, in which a significant decrease in this parameter was observed (Table 3). The percentage of muscle fat increased in the three groups—significantly in the EE_2_ group. When comparing the values among the three groups at this stage, the lowest muscle protein and fat values were observed in the EE_2_ group, even though these differences were not statistically significant (*p* > 0.05) (Table 3). The muscle textural values were significantly higher at this stage than in the previous stage in all the groups (*p* < 0.05). When comparing the three groups with each other at this stage, we could observe that the texture values were higher in the C group, whereas the EE_2_ and G1 groups showed the lowest values (*p* > 0.05) (Table 3).

At the end of the experiment, the percentage of muscle protein decreased in the C and G1 groups (Table 3). This decrease was significant in the G1 group in comparison with the values of this group at the beginning of the experiment. On the other hand, the percentage of muscle fat increased significantly in all the groups. When comparing the three groups with each other at this stage, we can observe that the percentages of muscle protein did not show significant differences among the three groups (Table 3). The percentage of muscle fat was higher in the C group, followed by the G1 group, while the EE_2_ group showed the lowest values (Table 3). At the end of the experiment, the muscle textural firmness increased in all the groups but not significantly (Table 3). When comparing the textural values among the three groups at this stage, these were found to be higher in the C group, followed by the G1 group. The EE_2_ group showed the lowest values (Table 3).

Taking all the data together at the end of the experiment, the correlation analysis showed a significantly negative correlation between the hardness values and the size of fibers (*r* = −0.547; *p* < 0.05), but a significantly positive correlation with the number and density of muscle fibers (*r* = 0.494; *p* < 0.05; and *r* = 0.624, *p* < 0.01, respectively). The fat content showed a significantly positive correlation with the HSI values (*r* = 0.683; *p* < 0.01), whereas the muscle protein content showed a significantly negative correlation with the HSI values (*r* = −0.656; *p* < 0.01), as well as with the muscle fat content (*r* = −0.94; *p* < 0.01).

#### Muscle Composition and Textural Hardness According to Gonad Structure

**Figure 7:**
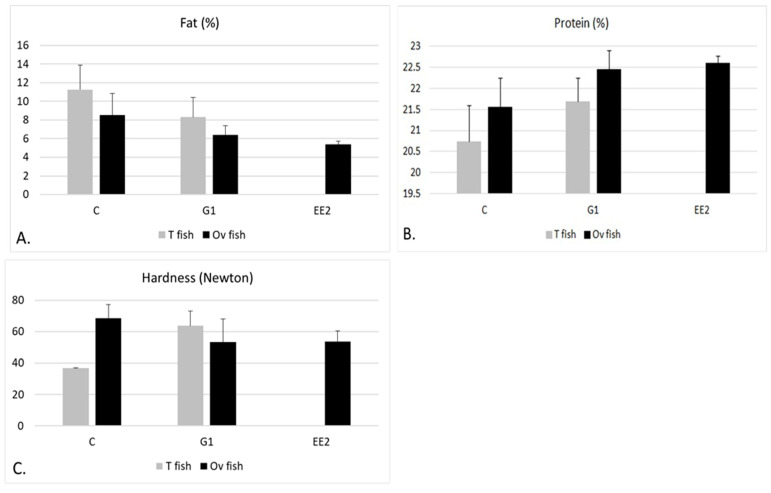
(**A**–**C**) Mean values (+SEM) of muscle fat, muscle protein and muscle hardness in T and Ov fish in the three groups at the end of the experiment. The fish studied in the EE_2_ group were 100% Ov fish at the end of the experiment and therefore there are no data for T fish in this group. No statistically significant differences were observed among the different groups.

When comparing the muscle composition of T fish in the C group versus the G1 group at the end of the experiment, the fat content was found to be higher in T fish in the C group (*p* > 0.05) (Figure 7A). On the other hand, when comparing the values of T and Ov fish in the three groups, the fat content was lower in Ov than in T fish, the EE_2_ group showing the lowest values (*p* > 0.05). Protein content showed the opposite tendency to that described for fat content (Figure 7B). In relation to the hardness values, these were lower in T fish in the C group than in T fish in the G1 group (Figure 7C)—an observation that is consistent with the size of the fibers of both groups, previously described (Figure 5A,B). When comparing the hardness values among the Ov fish in the three groups, the EE_2_ and the G1 groups showed the lowest values (*p* > 0.05).

### 2.4. Growth-Related Gene Expression

**Figure 8:**
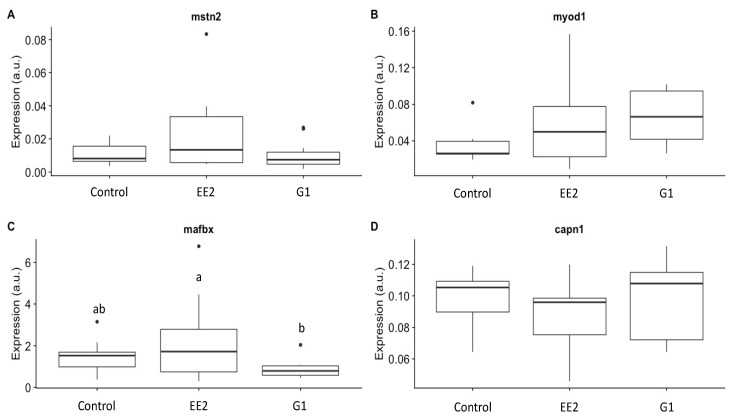
(**A**–**D**) Gene expression of *mstn2*, *myod1*, *mafbx* and *capn1*, respectively, in the C, EE_2_ and G1 groups. The values that are represented in the graphs for each gene correspond to the results that were obtained jointly in all stages for each experimental group. a.u: arbitrary units. In (**C**), different letters indicate significant differences among the experimental groups.

The study of gene expression throughout the experiment showed no statistically significant differences in the expression of *msnt2*, *myod1* and *capn1* genes among treatments (Figure 8). However, we did find significant differences in the expression of the *mafbx* gene. Thus, the EE_2_ group showed values statistically significantly higher than the G1 group (*p* = 0.03; Figure 8C).

**Figure 9:**
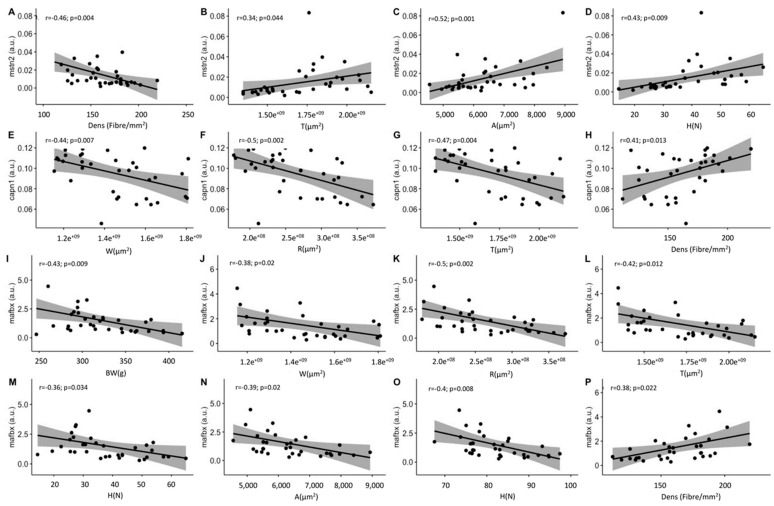
(**A**–**P**) Significant correlation plots between gene expression and muscle parameters from the three experimental groups (C, EE_2_ and G1). Pearson correlation index (*r*) and level of significance (*p*) are indicated for each plot. The values that are represented in the graphs correspond to the results that were obtained for each parameter considering all the stages of the experiment together. a.u: arbitrary units. BW: body weight (g); W: white muscle transverse area (µm^2^); R: red muscle transverse area (µm^2^); T: total transverse area of the myotome (µm^2^); A: area of white muscle fibers (µm^2^); D: minimum diameter of white muscle fibers (µm); H: hardness of the fillet (Newton); Dens: fibrillar density (number of muscle fibers mm^–2^).

The possibility of a correlation between the expression of the analyzed genes and the muscle parameters was also studied (Table 4; Figure 9). For instance, *mstn2* was positively correlated with total area (*r* = 0.34; *p* = 0.04), fiber area (*r* = 0.53; *p* = 0.001) and texture (*r* = 0.43; *p* = 0.009), while negatively correlated with density (*r* = −0.46; *p* = 0.004). The expression of *capn1* was negatively correlated with the number of white fibers (*r* = −0.44; *p* = 0.007), fiber density (*r* = −0.47; *p* = 0.004) and red muscle transverse area (*r* = −0.5; *p* = 0.002), while positively correlated with the total area (*r* = 0.41; *p* = 0.01) (Table 4; Figure 9). *Mafbx* was negatively correlated with body weight (*r* = −0.43; *p* = 0.009), number of white fibers (*r* = −0.38; *p* = 0.02), red muscle transverse area (*r* = −0.5; *p* = 0.002), total muscle area (*r* = −0.42; *p* = 0.01), hardness (*r* = −0.36; *p* = 0.03), fiber area (*r* = −0.39; *p* = 0.02) and muscle fiber diameter (*r* = −0.44; *p* = 0.008), while it was positively correlated with muscle fiber density (*r* = 0.38; *p* = 0.02) (Table 4; Figure 9).

## 3. Discussion

### 3.1. Body Growth and Survival

EE_2_ is a synthetic estrogen that causes alterations in reproductive capacity and sex differentiation in fish [5,6,7,8,9,10,11,12,13,16]. In previous studies, we observed that EE_2_ produced an endocrine disruption during the second and third reproductive cycles of gilthead seabream [22]. In the present study, we have exposed the specimens to EE_2_ during the second reproductive cycle for a period of time different to that used in the previous study. On the other hand, G1 is a G protein-coupled estrogen receptor (GPER1), which binds to E_2_ and other estrogenic compounds and therefore can produce rapid estrogen-mediated effects [39]. Interestingly, little is known about the effect of estrogenic compounds and the impact of GPER1 on fish growth.

As expected, body growth of the seabream specimens increased throughout the experiment in all groups. However, at 45 days of treatment (first stage) the BL values were significantly lower in the G1 group compared with the other two groups. After treatment cessation, in the second and third stages, the lowest body growth (BW, GW and BL) was observed in the EE_2_ group, whereas the G1 group improved its body growth, reaching similar values to those of the C group. These results seem to indicate a short and transitory catabolic effect of G1 treatment on body growth, while the influence of EE_2_ appears later and is persistent. In a previous study, we demonstrated the influence of EE_2_ on the immune response of gilthead seabream juveniles and it was observed that EE_2_ promoted a decrease in body weight (BW), which lasted throughout the recovery period (333 days after ceasing the treatments) [23]. On the contrary, G1 did not promote an evident estrogenic response on BW in the cited study. EE_2_ also inhibited the body growth in tilapia, *Oreochromis niloticus* [40], and zebrafish, *Danio rerio* [16]. However, zebrafish were able to compensate the delay in growth 40 days after the depuration of EE_2_ in clean water [16]. The catabolic effect of these synthetic estrogenic compounds is similar to that observed for natural estrogens in fish growth. Thus, in rainbow trout E_2_ produced a catabolic effect, reducing growth and fillet yield [24,41,42,43] by decreasing protein synthesis and increasing protein degradation.

In the present study, the EE_2_ and G1 treatments did not significantly influence HSI values. In contrast, an approximately 60% increase in HSI values was observed in rainbow trout specimens treated with E_2_ as a consequence of the increase in hepatic metabolic activity [44]. In a previous study [23], an increase of the HSI values in seabream juveniles was observed by us after 110 days of exposure to EE_2_, whereas such an increase was not observed in juveniles that were exposed to G1. However, in the present work the fish were only treated for 45 days and the EE_2_ and G1 in vivo exposure did not significantly influence HSI values.

At the beginning of the present experiment, the fish in all groups showed a predominance of the testicular area over the ovarian area in the gonads (100% T fish), corresponding with the first stage of the second reproductive cycle of this species. At the end of the experiment this proportion changed as expected in the last stages of the second reproductive cycle due to the proliferation of immature cells in the ovarian area [20]. In this work, variations were observed between experimental groups. Thus, all the fish that were studied in the EE_2_ group (6/6) showed a predominance of the ovarian area over the testicular area in the gonads (100% Ov fish), whereas the C and G1 groups showed 66.67% and 50% Ov fish, respectively. These results correlated with higher GSI values in Ov than in T fish in the present study but lower HSI values in Ov than in T fish, and these values were associated with lower BW, WB, BL and CF values in Ov fish in the experiment, the EE_2_ group (100% Ov fish) showing the lowest growth. The results of the present work suggest that EE_2_ treatment increases the growth of the ovarian area of the gonads and that this is associated with a redirection of nutrients to gonadal development and a decrease in growth rates, as observed in trout specimens that were treated with E_2_ [24]. Furthermore, although the fish in the present study were not undergoing a sex change but were only in a gonadal growth stage, it has been reported in previous studies that E_2_ induces feminization in older seabream [45,46]. Furthermore, several studies of salmonids have reported that fish reduce or cease food consumption during sexual maturation [47]. However, the data of the present work showed no differences in cumulative food intake between treated groups nor in the slope of the curves. These data suggest that the fish in this study did not change their food intake during the experimental period and that food intake was similar in all experimental groups. Similarly, EE_2_ and G1 treatments did not influence the dietary intake of seabream juveniles [23]. On the other hand, survival was lower in the EE_2_ group than in the G1 and C groups (83% in EE_2_ versus 100% in the G1 and C groups). However, in a previous study carried out at the same stage as the one studied in the present work (i.e., the second reproductive cycle) treatment with EE_2_ did not affect the survival of seabream [22]. This may be due to the fact that, while in the present work doses of 5 µg of EE_2_ per g food were applied for 45 days, in the aforementioned work [22] doses of 2.5 to 5 µg/g food were applied for 28 days. Given that the effect of these compounds depends on the dose, exposure time, age and stages of the reproductive cycle [6,7], it could be that the period of exposure to EE_2_ treatment in the present study was detrimental to survival at the dose and reproductive stage studied. In a previous study [6], mature gilthead seabream males were treated with 5, 50, 125 and 200 µg of EE_2_/g food and a correlation was found between time of exposure and dose of EE_2_, as with the highest doses the mortalities started earlier than with the lowest doses [6]. Hence, it is now necessary to delve into the effect of different doses of this compound at different exposure periods, ages and reproductive stages of the specimens.

### 3.2. Muscle Growth Parameters, Muscle Enzymatic Activity and Textural Hardness

Studies of the influence of steroids on the mechanisms of fish muscle growth are still scarce and have mainly been carried out on salmonids [4,24]. In addition, to the best of our knowledge, no studies have evaluated the effect of EE_2_ and GPER1 activation on the dynamic of fish muscle growth. Hence, the present study evaluates the influence of both compounds (EE_2_ and G1) in the muscle characteristics of seabream during sexual maturity (second reproductive cycle). Throughout the present experiment, the growth of W, R and the total myotome increased in all the groups. However, muscle growth was higher in the C group, followed by the G1 group, the EE_2_ group showing the lowest muscle growth in the second and third stages. This trend seems to show a catabolic effect of EE_2_ on muscle in the medium and long term, whereas this effect was observed to a lesser extent in G1-treated fish. As has been already mentioned, this catabolic effect has also been observed for E_2_ in salmonids [24,41]. E_2_ promotes protein catabolism in the muscle tissue of salmonids [48,49], supporting the notion that E_2_ may serve as a maturation-induced signal that initiates these effects. E_2_ reduces circulating concentrations of insulin-like growth factor-I (IGF-I) [50], an anabolic hormone that promotes growth by affecting multiple mitogenic and metabolic processes in muscle cells, including increasing protein synthesis and decreasing protein degradation [51,52,53]. Cross-talk between GPER1 and two main growth factor receptors, EGFR and insulin-like growth factor-I receptor (IGF-IR), has also been reported [54]. Numerous studies suggest that the complex interaction of these factors may contribute to the progression of diverse types of cancer [54].

Other authors have studied the physiology of female trout during sexual maturity and found that E_2_ caused a decrease in growth rates associated with a redirection of nutrients to gonadal development at the expense of muscle growth [24]. According to the results obtained by these authors, E_2_-induced reductions in myogenic gene expression (*fst* and *myog*) likely contribute to decreased muscle growth in part to direct nutrients from muscle growth toward maturation-related processes, such as gonadal development. Further support for this idea comes from the increased expression of cathepsin-L, which suggests increased protein degradation through the autophagy–lysosome system (ALS). Proteolytic mechanisms have previously been reported to increase with sexual maturation (43) and/or E_2_ treatment [41], which likely functions to mobilize nutrient stores.

The generation of fibers (hyperplasia) requires more energy than their hypertrophy [55] and implies a greater muscle growth potential than hypertrophy, since the fibrillar area has a physiological maximum [56,57]. When comparing muscle cellularity among the three groups throughout the present experiment, the highest mean hypertrophy values were found in the EE_2_ and G1 groups, whereas hyperplasia and muscle fiber density showed the opposite trend to that observed for hypertrophy. Therefore, these results reflect a lower muscle growth potential in the treated groups (EE_2_ and G1) than in the C group throughout the experiment. In addition, at the end of the experiment, some differences were observed between the Ov and T fish, hyperplasia being higher in the Ov than in the T fish in the C group, whereas the contrary tendency was observed for hypertrophy in this group. Furthermore, Ov fish in the C group showed a higher number of fibers than the other groups, thus showing the greatest potential growth.

Related to the hardness values of fillets, these increased in all groups throughout the experiment. When comparing the values among the different groups at each stage, we observed that the mean hardness values were lower in the EE_2_ and G1 groups than in the C group, a negative correlation being observed with respect to the size of the fibers, which is in agreement with other studies on teleost fish [36,37,38]. When comparing Ov versus T fish in the three groups at the end of the experiment, the lowest hardness values were observed in Ov fish belonging to the EE_2_ group (100% Ov fish) and the G1 group (50% Ov fish) and in the T fish in the C group (33.3% T fish). These values correlate with the size of the fibers of the cited groups.

Throughout the three stages of the experiment, larger fibers presented higher glycolytic activity than medium and small fibers. Since the EE_2_ and G1 groups presented more hypertrophy than the C group, the glycolytic potential of the treated groups was higher than that observed in the C group, although, as described above, the T fish in the C group also showed high hypertrophy values at the end of the experiment and, hence, high glycolytic activity. These results agree with previous findings in mammals showing higher glycolytic metabolism and an increased proportion of fast-twitch glycolytic fibers in the muscles of cows with muscular hypertrophy [58,59,60]. In beef (cattle meat) it has been observed that larger fibers (and, therefore, higher glycolytic activity) tend to deteriorate faster during postmortem degradation, probably due to greater activity of the proteolytic enzymes in the muscle (cathepsins and calpains) which simultaneously reduce firmness [61]. Thus, according to our results, the EE_2_ and G1 groups could present faster postmortem degradation associated with the larger size of fibers and their higher enzymatic activity. This, together with the lower values of textural firmness in the treated groups, may mean a lower quality of the final product in EE_2_- and G1-exposed fish, which must be taken into account by the producer in order to avoid economic and quality losses. At the end of the experiment, the highest quality would be found in the Ov fish in the C group. Other effects that have been observed in meats with greater hypertrophy are: lower water retention capacity, lower pH, lower amounts of collagen, different profiles of fatty acids (tending to be more saturated in more hypertrophic meats), less coloration, etc. [62,63]. However, more specific studies of all parameters that influence quality characteristics and postmortem degradation would be needed in order to understand the influence of the hormonal treatments on the final quality of the fillets of seabream.

### 3.3. Muscle Protein and Fat

Altogether, the percentage of muscle protein decreased throughout the experiment in all groups, whereas the percentage of muscle fat increased, thus showing an inverse correlation with the protein content (*r* = −0.94; *p* < 0.01). The decrease of muscle protein is normal during the sexual maturity of the fish, since food reserves must be mobilized towards gonadal development [42,43]. However, the decrease of muscle protein was not uniform among the groups. Thus, in the EE_2_ group, protein content significantly decreased in the second stage but was recovered at the end of the experiment. In contrast, muscle protein decreased in the C and G1 groups only at the end of the experiment. The fat values were lower in EE_2_ than in the other groups throughout the experiment. On the other hand, when comparing the fat and protein content of Ov and T fish in the three groups at the end of the experiment, the fat content was lower in Ov than in T fish, the EE_2_ group (100% Ov fish) showing the lowest values, whereas protein content showed the contrary tendency. In addition, as described previously, these results were parallel to low HSI and high GSI values in Ov fish at the end of the experiment. Gonadal development involving the mobilization of fat from the liver and muscles [42,43], these results seem to indicate that this is what is going on in Ov fish towards the end of the experiment, the results being more pronounced in the EE_2_ group.

### 3.4. Growth-Related Gene Expression

In the present work, the biological processes occurring in the skeletal muscle in response to EE_2_ or the G1 agonist were studied by analyzing some key genes. *Mafbx* was the only gene significantly affected by the treatments. *Mafbx* is a muscle-specific E3 ubiquitin ligase, part of the proteome network, which is upregulated in response to catabolic signals [64,65] and has been demonstrated to be upregulated also in response to an accute injection of E3 [24]. In the present work, *mafbx* expression was significantly higher in EE_2_ and it was correlated with the lowest values of BW, BL, W and R. Similarly, previous authors found that E_2_ promoted protein catabolism in rainbow trout by decreasing protein synthesis and increasing protein degradation [24]. In addition, *mstn2*, a known negative regulator of muscle growth [66], was higher in the EE_2_ group of the present study. On the other hand, *capn1* is part of the calpastin/calpain system and is also involved in fish muscle protein degradation [67]. In line with this, *capn1* in the present study was negatively correlated with growth and muscle structure parameters (Table 4). Correlations between *capn1*, *mafbx* and *mstn2* expression and fiber size and number suggest that *mstn2* might be inhibiting the formation of new fibers, while protein degradation is slowing muscle growth, which would explain the lower body weight of EE_2_ fish and the lower fiber density found in them as a consequence of a tendency to develop larger muscle fibers.

## 4. Material and Methods

### 4.1. Animals and Management

This research was carried out on healthy specimens of gilthead seabream (*Sparus aurata*) obtained from a broodstock of gilthead seabream bred at the aquaculture facilities of the Instituto Español de Oceanografía (IEO-CSIC) located in Mazarrón, Murcia, Spain. All the specimens were kept under the same culture conditions from hatching to the beginning of the feeding trial. Two-year-old gilthead seabream males at the spermatogenesis stage of the second reproductive cycle (*n* = 60; 272.3 ± 23.2 g average body weight ± SEM; 25.9 ± 0.7 cm average total body length ± SEM) were randomly distributed in three experimental groups in duplicate tanks (20 fish group^−1^) of 2 m^3^ tank^−1^; 10 fish tank^−1^. Initial stock density was 1.4 ± 0.1 kg m^−3^, and sea water renewal rate (37‰ salinity) was kept at 1000 L h^−1^ in an open flow circuit, maintaining values of ammonia and nitrites (<0.1 mg/L) suitable for gilthead seabream culture. SERA GmbH commercial tests were used for the quantification of ammonia and nitrites.

Animals were kept under natural photoperiod and temperature, so that the water temperature gradually increased from 14.9 ± 0.3 °C at the beginning of the feeding trial (January) to 25.0 ± 0.8 °C (June) during the assay, while the photoperiod initially was 9:15 h L:D and progressively changed to 14:10 h L:D. The light intensity ranged from 40–60 lux. The tanks were equipped with aerators to maintain an adequate level of oxygenation (above 6 mg/L). Oxygen measurements were performed with an oximeter (Handy Polaris OxyGuard International A/S, DK-3460 Birkerod, Danmark).

All specimens studied were handled in accordance with the Guidelines of the European Union Council (2010/63/EU), the Ethics Committee for Animal Experimentation of the Mazarrón aquaculture facilities of the IEO-CSIC (REGA: ES300261040017) and approval was received from the Ministry of Water, Agriculture and Environment of the Autonomous Community Region of Murcia (Spain; A13200101).

### 4.2. Experimental Treatments and Quantification of Cumulative Intake and Final Survival

The fish were fed with a standard commercial diet (44% protein, 22% lipids; Skretting, Spain) containing 5 μg EE_2_ or G1/g food. The EE_2_ (98% purity; Sigma, Missouri, MO, USA) or G1 (98% purity; Tocris, Bristol, UK) were incorporated using the ethanol evaporation method (0.3 L ethanol/kg of food) described by other authors [68]. The control diet was prepared in the same way without adding EE_2_ or G1. The fish were fed with the experimental diet for 45 days and then fed the standard commercial diet without EE_2_ or G1 for a further 122 days. The daily intake of each tank was recorded throughout all the experimental period (167 days). The fish were fed three times a day (at 09:00, 14:00 and 17:00) ad libitum until a maximum of 1% of the tank biomass every day. The amount of food was progressively increased, as did the body weight of the fish. The remaining food was weighed daily and the total cumulative intake of food, EE_2_ and G1 was calculated.

**Figure 10:**
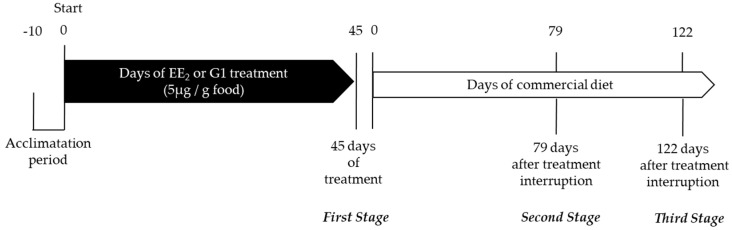
Schematic drawing of the experimental design. After 10 days of acclimatization, fish were subjected to three experimental conditions: control, EE_2_ or G1 treatment. In the EE_2_- and G1-treated groups, EE_2_ and G1 were incorporated into the commercial food. The control group (untreated fish) received the same commercial food without estrogenic compounds added. After 45 days, the EE_2_ and G1 treatments were interrupted. From that moment, the three experimental groups received a commercial diet. The samples were taken on day 45 of treatment (first stage) and 79 and 122 days after the cessation of the treatments (second and third stage, respectively) in the three groups.

Thus, three experimental groups were established: fish fed with a control diet (C group), fish fed with a diet containing EE_2_ (EE_2_ group) and fish fed with a diet containing G1 (G1 group). Before sampling, fish were fasted for 24 h. The experiment was performed after the final fattening of seabream specimens up to commercial size (final average body weight of 426.0 ± 47.2 g) in order to check the effects on final body and muscle growth as well as on the characteristics of the fillets. The experiment lasted 167 days, the three groups kept separately in order to study the immediate and lasting effect of these treatments (Figure 10). The survival percentage was also calculated for each group at the end of the experiment.

### 4.3. Sampling and Measurement of Body Parameters

Samples were taken 45 days after the application of the experimental treatments (first stage of study) and after 79 and 122 days of treatment cessation (second and third stages, respectively), from six seabream specimens per group (C, EE_2_ and G1 groups) at each sampling point (Figure 10). Thus, 18 fish were studied in each group (54 fish in total). The three stages correspond with the second reproductive cycle in which the specimens are functional males but with a certain proportion of immature ovarian area, which varies throughout this second cycle [7]. Thus, even though the population does not differ between functional males and females until the third reproductive cycle, the proportion of testicular and ovarian areas of the gonads changed in the different groups throughout the study, so we differentiated between specimens with higher proportions of ovarian area in the gonads (Ov fish—fish in which ovarian growth is more intensively induced) and specimens with the highest proportions of testicular area in the gonads (T fish). In the present work, the different proportions of testicular and ovarian areas was macroscopically analyzed at each sampling point. Furthermore, the body and muscle parameters, enzymatic muscle activity, muscle composition and textural hardness were analyzed according to the predominant gonadal area observed.

All fish from each group and at each stage were collected and slaughtered by an overdose of anesthesia with 40 ppm of clove oil. In each sampling point, the following body parameters were measured: body length (BL); body weight (BW); gutted weight (weight of the fish once the guts were removed) (GW); condition factors (CF) (g cm^−3^): 100 × (body weight/length^3^); hepatosomatic index (HSI) (%): 100 × liver weight/total weight; gonadosomatic index (GSI) (%): 100 × gonad weight/total weight. Subsequently, the fish were delivered to the University of Murcia for the analysis of the muscle parameters.

### 4.4. Quantitative Analysis of Muscle Growth and Histochemical Techniques

After measuring the body parameters of the specimens, these were cut transversely to the long body axis, and 5-mm thick whole body slices were obtained in the Faculty of Veterinary Medicine of the University of Murcia. The whole muscle cross-sections from each fish were photographed for further morphometric analysis. Subsequently, these body slices were cut into smaller blocks and then snap-frozen in 2-methylbutane over liquid nitrogen. Later, sections of 8 μm thickness were obtained from those frozen blocks in a cryostat (Leyca CM 1850, Leica Microsistemas SLU. Barcelona, Spain) and these sections were stained with haematoxylin-eosin and with nicotinamide adenine dinucleotide (reduced) tetrazolium reductase (NADH-TR) for morphometric and histochemical studies, respectively, of the muscle under light microscope. Muscle growth was quantified using an image analysis program for morphometric studies (Sygma-Scan Pro_5 system, Systat Software Inc. San Jose, California, CA, USA). The total cross-sectional area of the white and red muscle was measured in all the stages. In addition, the following parameters were measured: the number of white muscles fibers (N), the area (A) and minimum diameter of white muscle fibers (D) and muscle fiber density (number of white fibers μm^–2^) (Dens). The average size was estimated from ~600 fibers (±10 sd) located at the intermediate and apical sectors of the epaxial quadrant of the transversal section of the myotome, according to the methodology described in previous studies on fish [69,70].

The metabolic activity of white muscle fibers was also studied in the three groups throughout the experiment. For these analyses, the muscle sections, obtained from all fish as described above, were stained according to the technique described by the authors of [71] and subsequently modified by another author [72]. This technique detects the enzyme nicotinamide adenine dinucleotide (reduced) tetrazolium reductase (NADH-TR) and allows the oxidative (aerobic) and glycolytic (anaerobic) activity of the muscle fibers to be determined. With this technique, the mitochondria are stained blue, while the myofibrils are not stained. Thus, the red muscle of the fish (with oxidative metabolism) will be stained blue, while the white muscle (with glycolytic metabolism) will not be stained blue, even though the medium and small white fibers (more immature than the big fibers) will appear to have a slight blue tone due to the fact that they still have some oxidative capacity.

### 4.5. Analysis of the Composition and Texture of the Fillet

The content of muscle protein and fat was analyzed by the methods of Kjeldahl and Soxhlet, respectively, according to AOAC methods [73]. Textural measurements were carried out by compression, using a TA-XT2i Texture Analyser (Stable Micro Syss) equipped with a 25-kg load cell and a flat ended 10-mm-diameter cylindrical probe. The instrument’s software was Texture Expert Version 1.22. The pre-test speed, test speed and post-test speed were 1.5, 1.0 and 10 mm/s, respectively, deformed to 40% of original height. The data acquisition rate was 200 pps. The distance, maximum force and maximum shear force values obtained from the texture profile curve of each sample were used to calculate the hardness (the mechanical parameter that indicates the resistance to compression), following the methodology described by other authors [74]. Measurements were made for each muscle sample perpendicularly to the orientation of muscle fibers. The size of the muscle pieces was 4 cm width, 4 cm length and 2 cm height. For each sampling point, two muscle pieces were measured and then mean values were obtained.

### 4.6. Gene Expression Analysis

Total RNA was extracted from the white muscles of control or treated fish (n = 6 fish/treatment/time point) with TRIzol reagent (Invitrogen), following the manufacturer’s instructions, and quantified with a spectrophotometer (NanoDrop, ND-1000). The RNA was treated with DNase I, amplification grade (1 U/mg RNA; Invitrogen), to remove genomic DNA traces that might interfere with the PCRs, and the SuperScript IV RNase H reverse transcriptase (Invitrogen) was used to synthesize first-strand cDNA with oligo-dT18 primer from 1 µg total RNA, at 50 °C for 10 min. The b-actin (actb) gene was analyzed by semiquantitative PCR using an Eppendorf Mastercycle Gradient Instrument (Eppendorf). Reaction mixtures were incubated for 2 min at 95 °C, followed by 35 cycles of 45 s at 95 °C, 45 s at the specific annealing temperature, 1 min at 72 °C, and finally 10 min at 72 °C. The expression of *capn1*, *mafbx*, *mstn2* and *myod2* genes were analyzed by real-time RT-PCR performed with a QuantStudioTM 5 Flex instrument (Applied Biosystems, Waltham, Massachusetts, MA, USA) using SYBR Green PCR core reagents (Applied Biosystems). Reaction mixtures were incubated for 10 min at 95 °C, followed by 40 cycles of 15 s at 95 °C, 1 min at 60 °C and finally 15 s at 95 °C, 1 min at 60 °C, and 15 s at 95 °C. For each mRNA, gene expression was corrected by *ef1α* gene content in each sample using the comparative cycle threshold method (2^−ΔΔCt^). The gilthead seabream-specific primers used are shown in Table 5. In all cases, each PCR was performed with three technical replicates.

### 4.7. Statistical Analysis

The statistical analysis was performed with Statistical Package SPSS 24. The mean and standard error of the mean (SEM) were calculated for all the muscle parameters in each group at all the stages of the experiment. Data distribution and homogeneity of variances were analyzed using the Shapiro–Wilk and the Levene test, respectively. For most of the muscle parameters, the values of both tests showed *p*-values > 0.05, so the analysis of variance (ANOVA) and a post hoc Tukey test were used, with *p* < 0.05. In the other cases, non-parametric tests (Mann–Whitney U test, Kolmogorov–Smirnov Z) were used. Pearson correlation analysis was also performed for the different parameters throughout the experiment. Correlation analysis between gene expression and muscle parameters was carried out using the R-based function cor.test. Density functions for fiber area distributions were created in R-Studio using the ggplot2 package [75]. Statistical analysis of the density distribution of the muscle fibers was carried out using a Kolmogorov–Smirnov test.

## 5. Conclusions

1. GPER1 activation and EE_2_ treatment reduced body growth in the short and long term, respectively.
2. EE_2_ treatment, and to a lesser extent G1 treatment, reduced muscle growth (transverse area of the muscle) in the long term and was correlated with a high expression of key growth muscle-related genes in the EE_2_ group, such as *mfbx* (upregulated gene in catabolic signals) and *mstn2* (negative regulator gene of the muscle growth).
3. EE_2_ treatment and GPER1 activation increased the hypertrophy and glycolytic activity of the white muscle fibers, whereas fibrillary hyperplasia and muscle fiber density showed the contrary tendency, indicating immediate and persistent long-lasting effects on muscle cellularity.
4. EE_2_ treatment and GPER1 activation reduced the textural firmness, showing a negative correlation with the size of the muscle fibers.
5. EE_2_ exposure reduced muscle fat and was related with the predominance of ovarian area in the gonads of EE_2_-treated fish.

## Figures and Tables

**Table 1:** Mean values of body parameters of the three experimental groups throughout the three stages of the experiment. Different lowercase superscripts among groups within each stage indicate significant differences (*p* < 0.05) for each parameter. C, EE_2_, G1: control, EE_2_ and G1 groups, respectively; BW: body weight; GW: gutted weight; BL: body length; CF: condition factor; HSI: hepatosomatic index; GSI: gonadosomatic index. Values are expressed as mean ± SEM.

Stage	Groups	BW (g)	GW (g)	BL (cm)	CF (100 × g cm^−3^)	HSI (%)	GSI (%)
First (on day 45 of treatment)	C	314.4 ^a^ ± 8.5	286.86 ^a^ ± 7.41	26.6 ^a^ ± 0.2	1.68 ^ab^ ± 0.1	2.01 ^a^ ± 0.08	0.29 ^a^ ± 0.06
	EE_2_	292.7 ^a^ ± 8.76	265.8 ^a^ ± 7.8	26.75 ^a^ ± 0.2	1.53 ^b^ ± 0.01	2.53 ^a^ ± 0.22	0.26 ^a^ ± 0.1
	G1	288.6 ^a^ ± 6.41	262.8 ^a^ ± 5.98	25.67 ^b^ ± 0.3	1.71 ^a^ ± 0.05	2.4 ^a^ ± 0.06	0.19 ^a^ ± 0.04
Second (79 days after treatment cessation)	C	374.9 ^a^ ± 9.85	344.2 ^a^ ± 9.54	28.42 ^a^ ± 0.47	1.6 ^a^ ± 0.06	1.5 ^a^ ± 0.3	0.22 ^a^ ± 0.04
	EE_2_	300.23 ^b^ ± 12.16	292.6 ^b^ ± 7.12	27.17 ^b^ ± 0.21	1.5 ^a^ ± 0.08	1.87 ^a^ ± 0.57	0.2 ^a^ ± 0.03
	G1	366.03 ^a^ ± 9.09	336.2 ^a^ ± 9.18	27.92 ^ab^ ± 0.2	1.7 ^a^ ± 0.03	1.28 ^a^ ± 0.06	0.23 ^a^ ± 0.03
Third (122 days after treatment cessation)	C	428.23 ^ab^ ± 8.85	397.5 ^ab^ ± 8.24	29.0 ^a^ ± 0.36	1.76 ^a^ ± 0.05	0.93 ^a^ ± 0.05	0.22 ^a^ ± 0.05
	EE_2_	384.38 ^b^ ± 13.66	355.47 ^b^ ± 12.99	28.5 ^a^ ± 0.22	1.66 ^a^ ± 0.04	0.91 ^a^ ± 0.004	0.22 ^a^ ± 0.03
	G1	471.57 ^a^ ± 23.7	436.9 ^a^ ± 21.91	29.6 ^a^ ± 0.42	1.82 ^a^ ± 0.05	0.95 ^a^ ± 0.05	0.18 ^a^ ± 0.02

**Table 2:** Mean muscle parameter values in the three groups throughout the three stages. Different lowercase superscripts among groups within each stage indicate statistically significant differences (*p* < 0.05) for each parameter. C, EE_2_, G1: control, EE_2_ and G1 groups, respectively; W, R: transverse area of the white and red muscles, respectively; A: area of white muscle fibers; D: minimum diameter of white muscle fibers; N: number of white muscle fibers; Dens: fibrillar density (number of muscle fibers mm^–2^). Values are expressed as mean ± SEM.

Stage	Groups	W (mm^2^)	R (mm^2^)	A (μm^2^)	D (μm)	N	Dens
First (on day 45 of treatment)	C	1251.2 ^a^ ± 35.6	204.5 ^a^ ± 9.6	5246.52 ^a^ ± 248.5	74.5 ^a^ ± 2.28	241,808.4 ^a^ ± 15,472.2	192.64 ^a^ ± 8.6
	EE_2_	1327.1 ^a^ ± 49.2	224.75 ^a^ ± 9.4	5676.8 ^a^ ± 137.5	77.2 ^a^ ± 0.85	233,501.89 ^a^ ± 4373.5	176.69 ^a^ ± 4.46
	G1	1281.9 ^a^ ± 25.5	223.96 ^a^ ± 8.4	5773.6 ^a^ ± 265.4	78.8 ^a^ ± 1.73	223,892.97 ^a^ ± 9215.5	174.9 ^a^ ± 7.43
Second (79 days after treatment cessation)	C	1632.32 ^a^ ± 38.46	307.69 ^a^ ± 20.04	6825.29 ^a^ ± 440.66	85.40 ^a^ ± 3.2	244,515.14 ^a^ ± 17,359.1	149.7 ^a^ ± 9.8
	EE_2_	1580.99 ^a^ ± 50.36	287.15 ^a^ ± 11.44	6871.26 ^a^ ± 492.99	85.43 ^a^ ± 2.9	236,680.22 ^a^ ± 19,501.24	149.03 ^a^ ± 9.9
	G1	1608.37 ^a^ ± 67.2	318.86 ^a^ ± 10.75	7266.59 ^a^ ± 314.0	88.3 ^a^ ± 1.95	223,948.69 ^a^ ± 14,958.87	138.87 ^a^ ± 5.85
Third (122 days after treatment cessation)	C	2166.78 ^a^ ± 62.8	377.6 ^a^ ± 18.5	6853.4 ^a^ ± 526.3	82.4 ^a^ ± 3.4	323,080.1 ^a^ ± 20,136.3	149.8 ^a^ ± 10.2
	EE_2_	1924.18 ^a^ ± 80.9	340.3 ^a^ ± 10.7	8813.9 ^a^ ± 994.3	96.0 ^a^ ± 5.7	235,558.5 ^a^ ± 34,306.7	120.4 ^a^ ± 12.78
	G1	2063.9 ^a^ ± 90.1	374.6 ^a^ ± 12.9	7916.96 ^a^ ± 216.8	89.5 ^a^ ± 1.19	262,117.11 ^a^ ± 15,718.8	126.8 ^a^ ± 3.4

**Table 3:** Results for muscle protein and fat and for textural firmness of fillets (Hardness, N: Newton) from the three experimental groups of gilthead seabream throughout the three stages of the experiment. Different lowercase superscripts among groups within each stage indicate significant differences (*p* < 0.05) for each parameter. C, EE_2_, G1: control, EE_2_ and G1 groups, respectively. Values are expressed as mean ± SEM.

Stage	Groups	Protein (%)	Fat (%)	Hardness (N)
First (on day 45 of treatment)	C	22.77 ^a^ ± 0.29	1.63 ^ab^ ± 0.1	29.42 ^a^ ± 1.7
	EE_2_	22.50 ^a^ ± 0.33	1.28 ^b^ ± 0.04	29.21 ^a^ ± 1.14
	G1	22.30 ^a^ ± 0.42	1.67 ^a^ ± 0.12	23.87 ^a^ ± 2.43
Second (79 days after treatment cessation)	C	22.45 ^a^ ± 0.14	3.52 ^a^ ± 0.59	49.29 ^a^ ± 2.78
	EE_2_	21.87 ^a^ ± 0.2	2.93 ^a^ ± 0.36	46.7 ^a^ ± 2.55
	G1	22.38 ^a^ ± 0.34	3.47 ^a^ ± 0.33	47.14 ^a^ ± 4.87
Third (122 days after treatment cessation)	C	21.3 ^a^ ± 0.51	9.43 ^a^ ± 1.73	58.06 ^a^ ± 8.61
	EE_2_	22.6 ^a^ ± 0.17	5.38 ^a^ ± 0.37	53.8 ^a^ ± 6.64
	G1	22.03 ^a^ ± 0.31	7.18 ^a^ ± 1.1	56.16 ^a^ ± 6.93

**Table 4:** Correlation values of the genes with muscle growth parameters and muscle composition throughout the three stages considering the data for the three groups (C, EE_2_ and G1) together. BW: body weight; BL: body length; W: white muscle transverse area; R: red muscle transverse area; T: total transverse area of the myotome; A: area of white muscle fibers; D: minimum diameter of white muscle fibers; Prot: muscle protein; Fat: muscle fat; N: number of white muscle fibers; H: hardness of the fillet; Dens: fibrillar density (number of muscle fibers mm^–2^). Values are expressed as mean ± SEM. Significant differences are indicated as follows: · *p* < 0.1; * *p* < 0.05; ** *p* < 0.01).

Genes	BW	BL	W	R	T	Fat	Prot	H	A	D	N	Dens
*mstn2*	0.1	0.1	0.3 ·	0.4	0.3 *	0.3 ·	−0.3	0.4 **	0.5 **	0.4 **	−0.2	−0.5 **
*capn1*	−0.3 ·	−0.3 ·	−0.4 **	−0.5 **	−0.5 **	−0.1	−0.1	−0.2	−0.3 *	−0.3 *	0	0.4 *
*mafbx*	−0.5 **	−0.3 ·	−0.4 *	−0.5 **	−0.4 *	−0.2	−0.2	−0.4 *	−0.4 *	−0.4 **	−0.4 *	0.4 *
*myod*	−0.2	−0.2	−0.3 ·	−0.2	−0.3 ·	−0.2	0.1	−0.2	−0.1	−0.1	−0.2	0.1

**Table 5:** Forward and reverse primer sequences (5′–3′), ensemble accession number (ID), melting temperature of the amplicon (Tm), PCR efficiency (E) and amplicon product sizes in base pairs (bp). Genes are as follows: myoblast determination factor 2 (*myod2*), myostatin (*mstn2*), calpain 1 (*capn1*) and F-box protein 32 (*mafbx*).

Gene	ID	Primers (5′–3′)	Tm (°C)	E (%)	Size (bp)
*myod2*	ENSSAUG00010004893	F:CACTACAGCGGGGATTCAGAC	83.3	99	149
		R:CGTTTGCTTCTCCTGGACTC			
*mstn2*	ENSSAUG00010004791	F:ACCTGGTGAACAAAGCCAAC	84.8	99	201
		R:TGCGGTTGAAGTAGAGCATG			
*capn1*	ENSSAUG00010025995	F:CCTACGAGATGAGGATGGCT	87	95	114
		R:AGTTGTCAAAGTCGGCGGT			
*mafbx*	ENSG00000156804	F:GGTGCAACTTTCTGGGTTGT	85	97	105
		R:GGTCACCTGGAGTGGAAGAA			

## Data Availability

Not applicable.
